# Chromosomal imbalances in primary and metastatic pancreatic carcinoma as detected by interphase cytogenetics: basic findings and clinical aspects.

**DOI:** 10.1038/bjc.1998.223

**Published:** 1998-04

**Authors:** N. Zojer, M. Fiegl, L. MÃ¼llauer, A. Chott, S. Roka, J. Ackermann, M. Raderer, H. Kaufmann, A. Reiner, H. Huber, J. Drach

**Affiliations:** First Department of Internal Medicine, University of Vienna, Austria.

## Abstract

**Images:**


British Journal of Cancer (1998) 77(8), 1337–1342
© 1998 Cancer Research Campaign

### Chromosomal imbalances in primary and metastatic pancreatic carcinoma as detected by interphase cytogenetics: basic findings and clinical aspects

N Zojer[1], M Fiegl[1], L Müllauer[2], A Chott[2], S Roka[3], J Ackermann[1], M Raderer[1], H Kaufmann[1], A Reiner[4], H Huber[1] and J Drach[1]

[1]First Department of Internal Medicine, Division of Clinical Oncology, [2]Department of Clinical Pathology and [3]Department of Surgery, University of Vienna; [4]Department of Pathology, Donauspital, Vienna

**Summary** To date, cytogenetic studies on pancreatic carcinoma are rare, and little is known about the frequency of cytogenetic aberrations in primary carcinomas compared with metastatic tumour cells. We therefore evaluated the frequency of chromosomal aberrations in 12 primary pancreatic carcinomas and in effusion specimens from 25 patients with pancreatic cancer by using interphase fluorescence in situ hybridization (FISH) and a panel of four centromeric probes. Hyperdiploidy and chromosomal imbalances, predominantly affecting chromosome 8, were a constant finding in metastatic effusion cells, whereas concordant gain of chromosomes or relative loss of chromosome 18 characterized primary pancreatic carcinomas. The potential role of oncogenes located on chromosome 8 for pancreatic cancer progression was further investigated by double-hybridization studies of aneuploid effusion cells with a probe to 8q24 (MYC) and a centromeric probe to chromosome 8, which demonstrated amplification of the *MYC* oncogene in two of ten cases (20%). Finally, a potential application of basic findings in the clinical setting was tested by searching for micrometastatic cells in effusions from pancreatic cancer patients primarily negative by FISH. Two-colour FISH in combination with extensive screening (>10 000 nuclei) seems to be a useful tool to unequivocally identify micrometastatic cells by demonstrating hyperdiploidy and intranuclear chromosomal heterogeneity.

**Keywords:** interphase cytogenetics; pancreatic carcinoma; aneuploidy; micrometastasis detection

So far, 90 exocrine and endocrine pancreatic cancers have been karyotyped successfully (larger series on exocrine pancreatic carcinoma: Johansson et al, 1992; Bardi et al, 1993; Griffin et al, 1994, 1995; smaller series or case reports on exocrine or endocrine pancreatic tumours: van der Riet-Fox et al, 1979; Bullerdiek et al, 1985; Casalone et al, 1987; Teyssier, 1987; Scappaticci et al, 1992; Bardi et al, 1994; Bugalho et al, 1994; Danner et al, 1994; Long et al, 1994; Gorunova et al, 1995; Wiley et al, 1995; Grant et al, 1996). Considering the data available, loss of chromosome 18 is the most common numerical aberration identified by metaphase cytogenetics, occurring in half of the exocrine tumours with an abnormal karyotype. By comparative genomic hybridization, Solinas-Toldo et al (1996) demonstrated loss on 18q in 3 of 27 exocrine pancreatic cancers and Fukushige et al (1997) in five of six primary carcinomas and 10 of 12 cell lines. Recently, the tumour-suppressor gene *DPC4* was identified as the primary target of these aberrations (Hahn et al, 1996).

Besides chromosome 18, numerical aberrations in exocrine pancreatic tumours were reported to frequently involve chromosomes 7, 11, 12 and 20. Fluorescence in situ hybridization (FISH) with chromosome-specific probes can be used to visualize chromosomal aberrations of individual nuclei from paraffin-embedded and methanol–acetic acid-fixed material, thus allowing retrospective analysis of archived material. To delineate numerical chromosomal status of pancreatic carcinomas and to identify chromosomal patterns associated with pancreatic tumour progression, we performed double-hybridization experiments with a panel of four centromeric probes (chromosomes 7, 8, 11 and 18) in 12 primary pancreatic carcinomas and effusion specimens from 25 patients with pancreatic cancer. Gain of chromosomes 7 and 11 and loss of chromosome 18 are frequent findings by metaphase karyotyping in exocrine pancreatic tumours, thus providing the background for FISH analysis of these chromosomes. As chromosome 8 is frequently aberrant in primary and metastatic breast cancer (Roka et al, 1998) and plays a role in prostate cancer progression (Jenkins et al, 1997), we additionally selected a centromere-specific probe to this chromosome to analyse numerical aberrations of chromosome 8 and their potential significance for pancreatic cancer progression. In addition to pancreatic adenocarcinomas, four endocrine tumours of the pancreas were analysed by interphase FISH.

Finally, the potential impact of aneuploidy detection by FISH for the identification of pancreatic (micro-)metastatic cells in effusions was evaluated, using an approach previously performed in breast cancer effusions (Roka et al, 1998; Zojer et al, 1997).

*Received 31 July 1997*
*Revised 1 September 1997*
*Accepted 16 September 1997*

*Correspondence to:* J Drach, University of Vienna, Department of Internal Medicine I, Division of Clinical Oncology, Währinger Gürtel 18–20, A-1090 Vienna, Austria

### MATERIALS AND METHODS

### Clinical material

Paraffin-embedded tissue sections from 16 patients undergoing surgery for pancreatic tumours (1986–1995) were obtained from the Department of Clinical Pathology (University of Vienna) or the Department of Pathology of the Vienna Donauspital. These specimens comprised eight ductal adenocarcinomas, three periampullary carcinomas, one mucinous cystadenocarcinoma of the pancreas and four endocrine tumours of the pancreas (one gastrin, one insulin, one glucagon and one non-secretory tumour).

Preparation of the primary tumours followed the protocol detailed by Ott et al (1997). Briefly, 20-μm sections were cut from paraffin-embedded tissue blocks, dewaxed in xylene and rehydrated in graded alcohols. Subsequently, single-cell suspensions were obtained by digestion with 1 mg of proteinase XXIV (Sigma, Deisenhofen, Germany) in 2 ml of Carlsberg solution (0.1 M Tris buffer, 0.07 M sodium chloride, pH 7.2) for 1 h and then dropped onto a glass slide. To enhance probe accessibility to the nucleus, cells were incubated at 80°C in 1 M sodium thiocyanate and at 37°C in 0.4% Pepsin (in 0.2 N hydrochloric acid) for 1 and 3 min respectively.

Cells from 22 ascitic and three pleural effusions from patients with pancreatic cancer were gained by centrifugation of native effusion specimens, washed twice in phosphate-buffered saline, fixed in methanol–acetic acid (3:1) and stored at −80°C.

### FISH procedure and microscopy

The FISH protocol followed the standard procedure in our laboratory, as described in detail in a previous report (Drach et al, 1995). Directly, fluorescence-labelled alpha satellite probes (Vysis, Downers Grove, IL, USA), specific for the centromeric regions of human chromosomes 7, 8, 11 and 18, were applied. Two-colour FISH was performed using Spectrum Green-labelled probes in combination with Spectrum Orange-labelled probes, with chromosome 18 always being a partner in these combinations.

At least 200 non-overlapping nuclei from each effusion specimen and at least 100 nuclei from each primary tumour were evaluated by fluorescence microscopy (Olympus AH-3 microscope). Photographic documentation was performed using a Kodak Ektachrom 1600 film. In addition, images were acquired using a cooled, charged, coupled device (CCD) camera (Photometrics, Tucson, AZ) mounted on a Zeiss-Axioplan-2 immunofluorescence microscope and the Quips-XL FISH-imaging software (Vysis).

### Control specimens and criteria for true aneuploidy

Cut-off values for detection of true aneuploidy were calculated as mean signal numbers + three standard deviations of control cells from normal pancreatic tissue ($n = 1$) and chronic pancreatitis ($n = 2$) in the case of primary tumours; four effusion specimens from patients with non-malignant diseases served as controls for metastatic effusion cells.

Mean chromosome copy numbers for each tumour specimen and chromosome, which by definition are calculated by dividing the sum of the centromeric signals with the number of nuclei scored, are listed in Tables 1 and 2. Only nuclei aneuploid by two-colour FISH analysis were considered for calculation of mean copy numbers, and disomic cells (with a pattern of 2/2 signals for the Spectrum Green/ Spectrum Orange probe pair) were skipped from analysis. Concerning the nuclear status of 1/1-signal, 1/2-signal and 2/3-signal cells (or vice versa), only percentages of cells above cut-off were included in the calculation of mean copy numbers. For evaluation of malignant effusions, a cut-off for 3/4- and 4/4-signal cells was established, as a small population of mesothelial cells with this chromosomal pattern was found in control effusions (Fiegl et al, 1996).

### Definitions of modal ploidy and chromosomal imbalances

A modal ploidy status for a tumour specimen was determined if at least three of the four chromosomes showed mean copy numbers in the range of one ploidy unit, allocating the tumour to the corresponding ploidy category (triploidy, tri–tetraploidy, tetraploidy etc., see Tables 1 and 2). If all four chromosomes showed mean copy numbers in the range of one ploidy unit, no chromosomal imbalances were indicated (see Table 1). In the other case, deviation of the fourth chromosome was indicated as loss or gain. If less than three chromosomes showed mean copy numbers in the defined range, the chromosomal status was termed heterogeneous. Imbalances were predominant in these cases and a distinct ploidy was not evident. Similar criteria for characterization of ploidy and chromosomal imbalances were used in a FISH study of squamous cell carcinomas of the head and neck (Soder et al, 1995). Furthermore, we analysed three pancreatic carcinoma cell lines (BxPC-3, PANC-1, AsPC-1; all obtained from America Type Culture Collection (ATCC), Rockville, Maryland) using FISH and four centromeric probes. Ploidy estimated on the basis of the FISH results was in good agreement with the ploidy defined by metaphase karyotyping (data provided in the ATCC catalogue).

### MYC amplification in aneuploid effusions

In additional experiments, a Spectrum Orange-labelled probe to 8q24 (MYC) was used in combination with a Spectrum Green-labelled probe to the chromosome 8 centromere. Amplification of the *MYC* oncogene was defined as the presence of more than 20% of cells with over-representation of MYC signals in relation to chromosome 8 signals (Jenkins et al, 1997). At least 100 non-overlapping nuclei from each of the ten aneuploid effusion specimens were evaluated. Two effusion cell samples from patients with non-malignant diseases served as controls for the MYC studies.

### Micrometastatic cell detection

In effusion specimens with no detectable aneuploidy by the standard signal scoring procedure ($n = 15$), $1–2 \times 10^4$ nuclei (corresponding to 200 fields with 50–100 cells) were screened for the occurrence of rare aneuploid cells (as detailed previously; Roka et al, 1998). This is a procedure potentially practicable in the routine setting as, when in situ hybridization is appropriately performed (our laboratory set a minimum standard at 90% hybridization efficiency), it does not take more than 30 min to screen >10 000 nuclei. In this series, two-colour FISH with probe pairs of chromosomes 7/8 and 11/18 was performed, and rare grouped or single cells exhibiting more than four signals and concomitantly showing intranuclear chromosomal heterogeneity (e.g. a signal pattern of 5/2) were considered as unequivocal indicators of malignancy.

### RESULTS

### Chromosomal status of primary and metastatic pancreatic carcinoma

Significant differences were found by comparing the chromosomal status of primary carcinomas (neuroendocrine tumours were excluded from this comparative analysis) with that of metastatic specimens (Tables 1 and 2). A hyperdiploid modal ploidy status was observed in all primary carcinomas. Concordant gains of

© Cancer Research Campaign 1998

**Table 1** Chromosomal status of primary pancreatic carcinomas (P1–P12) and neuroendocrine tumours of the pancreas (P13–P16)

| No. | Histology[a] | Stage[b] | A (%)[c] | Ploidy | Chromosome copy number 7 | 8 | 11 | 18[d] | Imbalance |
|---|---|---|---|---|---|---|---|---|---|
| P1 | DA | T3N1M1 | 44.6 | Tri–tetr | 3.8 | 3.6 | 3.7 | 3.8 | – |
| P2 | DA | T3N1M0 | 38.2 | Tetr | 3.8 | 3.8 | 4.2 | 3.6m | – |
| P3 | DA | T3N1M0 | 20.7 | Tri–tetr | 3.8 | 3.1m | 3.5 | 3.6 | – |
| P4 | DA | T4N1M0 | 59.4 | Tri–tetr | 3.3 | 3.5 | 3.7 | 3.6 | – |
| P5 | PA | T2N0M0 | 24.4 | Tri | 3.1 | 3.0 | NSA | 2.4 | – |
| P6 | DA | T2N1M0 | 33.8 | Tri | 3.2 | 3.1 | 3.4 | 2.8 | – |
| P7 | MC | T2N0M0 | 23.0 | Het | 3.9 | 2.0 | 2.0 | 3.3 | Het |
| P8 | DA | T2N1M0 | 76.6 | Tetr | 4.2 | 2.5m | 3.8 | 3.5 | –8 |
| P9 | DA | T2N1M0 | 29.9 | Tetr | 4.2 | 3.7 | 4.1 | 6.4 | +18 |
| P10 | PA | T1NXM0 | 39.0 | Di–tri | 2.4 | 3.0 | 2.3 | 1.8M | –18 |
| P11 | PA | T2N0M0 | 46.3 | Tri–tetr | 3.4 | 3.8 | 3.3 | 2.1M | –18 |
| P12 | DA | T4N1M0 | 36.6 | Tri | 3.2 | 2.8 | 3.4 | 2.3 | –18 |
| P13 | NE (NS) | T2N0M0 | 75.6 | Het | 3.7 | 1.9m | 1.3m | 3.2 | Het |
| P14 | NE (GA) | T2N1M0 | 36.2 | Tri–tetr | 3.8 | 3.2 | 3.2 | 2.5m | –18 |
| P15 | NE (GL) | T1N1M0 | 61.0 | Tri | 3.1 | 2.8 | 1.9m | 2.6m | –11 |
| P16 | NE (IN) | T1NXM0 | 68.6 | Haploid | 3.6 | 1.5 M | 1.9m | 1.6 M | +7 |

[a]DA, ductal adenocarcinoma; PA, periampullary carcinoma; MC, mucinous cystadenocarcinoma; NE, neuroendocrine tumour; NS, non-secretory; GA, gastrinoma; GL, glucagonoma; IN, insulinoma; as classified by immunohistochemistry. [b]TNM classification of exocrine pancreatic tumours is also applied for neuroendocrine tumours of the pancreas. [c]Percentage of non-disomic cells (signal pattern not equal to 2/2). [d]Mean value of chromosome 18 copy numbers in three double-hybridization experiments. Het, heterogenous chromosomal status; m, subpopulation of tumour cells with monosomy for the respective chromosome; M, main population of tumour cells (>50%) exhibits monosomy for the respective chromosome; NSA, no significant aneuploidy.

**Table 2** Chromosomal status of malignant effusion cells from patients with pancreatic carcinoma (E1–E10) and comparison of FISH results with classification of effusions by cytological examination (E1–E25)

| No. | Site[a] | Cytology | A(%)[b] | Ploidy | Chromosome copy number 7 | 8 | 11 | 18[c] | Imbalance |
|---|---|---|---|---|---|---|---|---|---|
| E1 | A | Positive | 13.7 | Hex | 6.5 | 4.2 | 6.7 | 5.9 | –8 |
| E2 | A | Positive | 21.2 | Pent | 4.9 | 2.1 | 4.5 | 4.9 | –8 |
| E3 | P | Positive | 24.7 | Het | 3.5 | 4.7 | 3.9 | 2.1 | Het |
| E4 | A | Positive | 12.8 | Tri–tetr | 3.5 | 3.8 | 3.3 | 2.0 | –18 |
| E5 | A | Positive | 13.6 | Tri–tetr | 3.3 | 4.4 | 3.8 | 3.2 | +8 |
| E6 | A | Positive | 18.8 | Tetr | 3.4 | 5.6 | 4.4 | 4.2 | +8 |
| E7 | A | Positive | 73.5 | Tri | 3.2 | 4.2 | 3.3 | 3.1 | +8 |
| E8 | A | Negative | 10.6 | Tetr | NSA | 6.6 | 4.1 | 4.0 | +8 |
| E9 | A | Positive | 8.6 | Di–tri | 3.9 | NSA | NSA | 2.9 | +7 |
| E10 | P | Positive | 8.5 | Het | 5.6 | 3.7 | 2.4 | 2.9 | Het |
| E11 | A | Positive | | | Three islets (six aneuploid cells)[d] | | | | |
| E12 | A | Positive | | | Two islets (13 aneuploid cells)[d] | | | | |
| E13 | A | Positive | | | Five single aneuploid cells[d] | | | | |
| E14 | A | Negative | | | Two islets (23 aneuploid cells)[d] | | | | |
| E15 | A | Negative | | | Three islets (four aneuploid cells)[d] | | | | |
| E16–25 | 9A/1P | Negative | | | No aneuploidy | | | | |

[a]A, ascites; P, pleural effusion. [b]Percentage of non-disomic cells (signal pattern not equal to 2/2). [c]Mean value of chromosome 18 copy numbers in three double-hybridization experiments. Het, heterogenous chromosomal status; NSA, no significant aneuploidy. [d]Rare aneuploid cells as detected by extensive screening.

signal number for all chromosomes examined were found in 6 of 12 cases and no chromosomal imbalances were indicated in these tumours (P1–P6). In contrast, chromosomal imbalances were identified in all metastatic carcinoma specimens ($P < 0.01$, $\chi^2$-test), with chromosome 8 being predominantly affected (see Figure 1A).

Chromosome 8 imbalances were found in six of ten metastatic specimens in our series (four gains and two losses), a frequency (60%) that is significantly different ($P < 0.01$, $\chi^2$-test) to the frequency of chromosome 8 imbalances in primary carcinomas (1 of 12 specimens or 8%, see Table 1).

Monosomy 18 was observed in two primary pancreatic carcinomas (P10 and P11) that were resected at an early stage of tumour progression (TINXM0 and T2N0M0), which is indicative of loss of chromosome 18 being an early event in pancreatic carcinogenesis. In three other primary tumours, small subpopulations of cells with monosomy 8 or monosomy 18 were identified, as indicated in Table 1. However, no monosomic cells were found in the metastatic specimens.

In contrast, there was a trend towards higher chromosome copy numbers in metastatic disease. Ploidy of two effusion specimens (El and E2) was designated pentaploid and hexaploid, respectively, and gains of individual chromosomes, with mean copy numbers in the pentasomic to hexasomic range, were observed in three other effusions (E6, E8, E10).

© Cancer Research Campaign 1998

**Figure 1** (**A**) In effusion specimen E1, nuclei with five to seven signals for chromosome 18 (red) and three to four signals for chromosome 8 (green) are present, indicative of relative loss of chromosome 8 in this specimen (see also Table 1). (**B**) In nucleus (left) from effusion E2, MYC amplification is demonstrated by the presence of five red signals for 8q24 (MYC) in relation to only two signals for the centromere of chromosome 8 (green). Nucleus to the upper right shows normal signal pattern. (**C**) In effusion E11, rare aneuploid cells were detected by screening of >10 000 nuclei, as exemplified in this figure. Aneuploid nucleus with six signals for chromosome 7 (red) and five signals for chromosome 8 (green) is surrounded by a population of lymphocytes. Hyperdiploidy and concomitant intranuclear chromosomal heterogeneity indicates malignancy

### Chromosomal status of endocrine pancreatic tumours

Chromosomal status of endocrine tumours was different to that observed in adenocarcinomas. Chromosomal imbalances with monosomic cell populations were a feature of all four endocrine tumours studied (see Table 1). One tumour (N4) was designated haploid because of the predominance of nuclei with monosomy of chromosomes 8, 11 and 18. This finding is in line with a previous study (Long et al, 1994), in which near-haploid clones were identified in two endocrine neoplasms of the pancreas by metaphase karyotyping.

### MYC studies

Two out of ten malignant pancreatic effusion specimens (20%) showed MYC amplification, as defined in the Materials and methods section. High-level amplification, with a MYC–centromere 8 ratio >2, was observed in effusion E2 (in 32.6% of all nuclei counted, see Figure 1B), which additionally exhibited relative loss of chromosome 8 as indicated in Table 1. On the other hand, effusion specimen E7 was characterized by a relative gain of chromosome 8 in association with a low level of MYC amplification (with a MYC–centromere 8 ratio of 1.1–2 in 36.9% of nuclei).

© Cancer Research Campaign 1998

In the two control effusions, MYC was over-represented in relation to chromosome 8 in less than 2% of cells evaluated.

### FISH as a diagnostic tool to detect micrometastatic cells

We have shown previously that FISH using centromeric probes can improve malignant cell detection in effusions from breast cancer patients (Zojer et al, 1997). In the present study, we investigated whether or not this is also true for patients with pancreatic cancer by comparing the results of cytological examination of 22 ascitic and three pleural effusion specimens with the FISH results.

Twelve out of 25 effusion specimens (48%) were classified as malignant by cytological examination, whereas 15 of 25 (60%) were considered to be aneuploid, as analysed by FISH in a blinded fashion ($P$ = NS, $\chi^2$-test). Concordant classification by cytology and FISH was achieved in 22 of 25 cases. However, in three cytologically negative effusions, aneuploidy above background (E8) or rare aneuploid cells (E14, E15) could be detected by two-colour FISH. All samples with disomic results by FISH ($n = 10$) were also negative for malignant cells on cytological examination.

In effusions E11–E15, which were classified as disomic by FISH based on analysis of 200 cells, rare aneuploid cells and thus malignancy could be demonstrated by extensive screening (Figure 1C). In these cases, aneuploid nuclei were present as individual cells or in small tumour cell islets (with an estimated individualized frequency of 1:100–1:1000 reactive cells), showing gain of centromeric signals $>4$ and concomitant intranuclear heterogeneity, as required by definition.

### DISCUSSION

Numerical aberrations of chromosome 8 were only infrequently reported by metaphase karyotyping studies of exocrine pancreatic carcinomas. In our report, we show that relative loss or gain of the centromeric region of chromosome 8 (indicative of whole chromosome loss or gain respectively) may be a prominent feature of metastatic pancreatic carcinoma.

A region-specific probe to 8q24 (MYC) was selected to analyse chromosome 8 aberrations in metastatic effusions in more detail. To date, only few data on MYC amplification in pancreatic carcinoma are available. Yamada et al (1986) found amplification of the *MYC* oncogene in one primary pancreatic carcinoma, as well as in its metastasis, and Sakorafas et al (1995) reported expression of MYC by immunohistochemistry in two of ten cases. In our series, two effusions with chromosome 8 imbalance showed MYC amplification, whereas the other cases had concordant signal numbers with the centromeric and the 8q24-specific probes. Over-representation of MYC was thus demonstrated in five out of ten malignant pancreatic effusions, when the specimens with relative increase of chromosome 8 copy number were included (see Table 2).

Previous cytogenetic studies differ in the reported frequencies of gain of 8q in primary pancreatic carcinomas. Solinas-Toldo et al (1996) found gain of 8q in 3 of 27 primary tumours, whereas Fukushige et al (1997) reported gain of 8q in three of six primary tumours and 11 of 12 cell lines. Summarizing these results and the results from metaphase cytogenetic studies, gain of 8q appears to be part of the cytogenetic profile of at least some primary pancreatic carcinomas. Thus, MYC may already be over-represented in primary pancreatic carcinomas, and another locus may be the main target for numerical aberrations of chromosome 8 in metastasizing pancreatic cancer cells.

Besides aberrations of chromosome 8, metastatic pancreatic carcinomas are characterized by a generally higher frequency of chromosomal imbalances and higher ploidy designations compared with primary tumours. The more extensive genetic alterations in metastatic disease can be explained by mitotic malsegregation and endoreduplication, which continue to occur during solid tumour development, leading to accumulation of numerical chromosomal imbalances in the former case and increase in DNA content to hyperploidy in the latter (Dutrillaux et al, 1991). Endoreduplication, accompanying tumour progression, may also turn monosomy 18 of primary carcinomas into 'relative loss' of chromosome 18, which was indeed observed in one metastatic specimen (E4; relative loss of a chromosome means under-representation in relation to the defined ploidy, whereas monosomy indicates the presence of a single copy of this chromosome).

As chromosome 18 was reported to be frequently aberrant in metaphase cytogenetic studies of pancreatic carcinoma, this chromosome was targeted in all two-colour FISH experiments, excluding MYC studies and studies of micrometastasis detection. Four out of 22 pancreatic tumour specimens (primary tumours and effusion cell samples) exhibited relative loss of chromosome 18, a frequency (18%) lower than the frequency determined by metaphase karyotyping (Johansson et al, 1992; Bardi et al, 1993; Griffin et al, 1994, 1995) and approximating the results obtained by comparative genomic hybridization for loss of 18q (Solinas-Toldo et al, 1996; Fukushige et al, 1997). Loss of 18q may be one mechanism of inactivation of the recently identified tumour-suppressor gene *DPC4* (Hahn et al, 1996).

One particular focus of our work is to establish applications of FISH for diagnostic procedures in the clinical setting (Fiegl et al, 1995; Schenk et al, 1997; Zojer et al, 1997). In this study, we show that hyperdiploidy and intranuclear chromosomal heterogeneity are a constant finding in metastatic pancreatic cancer. This suggests that cohybridization with two centromeric probes may be a useful approach for unequivocal detection of rare (micro) metastatic cells, e.g. in effusions, peritoneal washings or bone marrow specimens.

We tested the implication of interphase FISH for malignant cell detection in effusions from patients with pancreatic cancer by comparing FISH results with data obtained by cytological examination. When extensive evaluation by screening of >10 000 nuclei from each effusion was used, FISH could detect rare aneuploid nuclei in three cytologically negative effusions, thus pointing to malignancy. As we demonstrated previously, FISH is a useful adjunct to cytological examination of effusions from breast cancer patients (Zojer et al, 1997). This also seems to be true for pancreatic cancer patients, although without statistical confirmation in this series.

Antibodies to cytokeratin are now commonly used for detection of micrometastatic cells in bone marrow of breast, colon and pancreatic cancer patients (Cote et al, 1991; Lindemann et al, 1992; Juhl et al, 1994). However, it was reported recently that some of these cytokeratin-positive bone marrow cells may actually be normal diploid cells of epithelial origin (Litle et al, 1997). We therefore propose to use FISH as a tool to unequivocally detect spread of pancreatic cancer to potential metastatic sites (e.g. peritoneal cavity, bone marrow), which may enhance further specification of prognostic subgroups of this disease.

© Cancer Research Campaign 1998

### ACKNOWLEDGEMENTS

This study was supported by a grant from the Austrian 'Fonds zur Förderung der wissenschaftlichen Forschung' (P-10893-MED) and 'Komission Onkologie'.

### REFERENCES

Bardi G, Johansson B, Pandis N, Mandahl N, Bak-Jensen E, Andren-Sandberg A, Mitelman F and Heim S (1993) Karyotypic abnormalities in tumours of the pancreas. *Br J Cancer* **67**: 1106–1112

Bardi G, Aman P, Johansson B, Pandis N, Mandahl N, Bak-Jensen E, Björkman A, Sjögren HO, Andren-Sandberg A, Mitelman F and Heim S (1994) Cytogenetic characterization of a periampullary adenocarcinoma of the pancreas, its liver metastasis, and a cell line established from the metastasis in a patient with Gardner's syndrome. *Cancer Genet Cytogenet* **76**: 29–32

Bugalho MJ, Roque L, Sobrinho LG, Hoog A, Nunes JF, Almeida JM, Leitao CN, Santos JR, Pereira MC and Santos MA (1994) Calcitonin-producing insulinoma: clinical, immunocytochemical and cytogenetical study. *Clin Endocrinol* **41**: 257–260

Bullerdiek J, Bartnitzke S, Kahrs E and Schloot W (1985) Further evidence for nonrandom chromosome changes in carcinoma cells – a report of 28 cases. *Cancer Genet Cytogenet* **16**: 33–43

Casalone R, Meriggi F, Forni E and Pasquali F (1987) Cytogenetic findings in a case of anaplastic carcinoma of the pancreas. *Cancer Genet Cytogenet* **29**: 253–259

Cote RJ, Rosen PP, Lesser ML, Old LJ and Osborne MP (1991) Prediction of early relapse in patients with operable breast cancer by detection of occult bone marrow micrometastases. *J Clin Oncol* **9**: 1749–1756

Danner DB, Hruban RH, Pitt HA, Hayashi R, Griffin CA and Perlman EJ (1994) Primitive neuroectodermal tumor arising in the pancreas. *Mod Pathol* **7**: 200–204

Drach J, Angerler J, Schuster J, Rothermundt CA, Thalhammer R, Haas OA, Jäger U, Fiegl M, Geissler K, Ludwig H and Huber H (1995) Interphase fluorescence in situ hybridisation identifies chromosomal abnormalities in plasma cells from patients with monoclonal gammopathy of undetermined significance. *Blood*, **86**: 3915–3921

Dutrillaux B, Gerbault-Seureau M, Remvikos Y, Zafrani B and Prieur M (1991) Breast cancer genetic evolution. I. Data from cytogenetics and DNA content. *Breast Cancer Res Treat* **19**: 245–255

Fiegl M, Tueni C, Schenk T, Jakesz R, Gnant M, Reiner A, Rudas M, Pirc-Danoewinata H, Marosi C, Huber H and Drach J (1995) Interphase cytogenetics reveals a high incidence of aneuploidy and intratumour heterogeneity in breast cancer. *Br J Cancer* **72**: 51–55

Fiegl M, Zojer N, Huber H and Drach J (1996) Hyperdiploidy in mesothelial cells from non-malignant effusions as detected by fluorescence in situ hybridization (FISH) (abstract). *Ann Hematol* **73** (suppl. II): A128, 512

Fukushige S, Waldman FM, Kimura M, Abe T, Furukawa T, Sunamura M, Kobari M and Horii A (1997) Frequent gain of copy number on the long arm of chromosome 20 in human pancreatic adenocarcinoma. *Gene Chromosome Cancer* **19**: 161–169

Gorunova L, Johansson B, Dawiskiba S, Andren-Sandberg A, Jin Y, Mandahl N, Heim S and Mitelman F (1995) Massive cytogenetic heterogeneity in a pancreatic carcinoma: fifty-four karyotypically unrelated clones. *Gene Chromosome Cancer* **14**: 259–266

Grant LD, Lauwers GY, Meloni AM, Stone JF, Betz JL, Vogel S and Andren-Sandberg A (1996) Unbalanced chromosomal translocation, der (17)t(13;17) (q14;p11) in a solid and cystic papillary epithelial neoplasm of the pancreas. *Am J Surg Pathol* **20**: 339–345

Griffin CA, Hruban RH, Long PP, Morsberger LA, Douna-Issa F and Yeo CJ (1994) Chromosome abnormalities in pancreatic adenocarcinoma. *Gene Chromosome Cancer* **9**: 93–100

Griffin CA, Hruban RH, Morsberger LA, Ellingham T, Long PP, Jaffee EM, Hauda KM, Bohlander SK and Yeo CJ (1995) Consistent chromosome abnormalities in adenocarcinoma of the pancreas. *Cancer Res* **55**: 2394–2399

Hahn SA, Schutte M, Shamsul Hoque ATM, Moskaluk CA, Da Costa LT, Rozenblum E, Weinstein CL, Fischer A, Yeo CJ, Hruban RH and Kern SE (1995) DPC44, a candidate tumor suppressor gene at human chromosome 18q21.1. *Science* **271**: 350–353

Jenkins RB, Qian J, Lieber MM and Bostwick DG (1997) Detection of MYC oncogene amplification and chromosomal anomalies in metastatic prostatic carcinoma by fluorescence in situ hybridization. *Cancer Res* **57**: 524–531

Johansson B, Bardi G, Heim S, Mandahl N, Mertens F, Bak-Jensen E, Andren-Sandberg A and Mitelman F (1992) Nonrandom chromosomal rearrangements in pancreatic carcinomas. *Cancer* **69**: 1674–1681

Juhl H, Stritzel M, Wroblewski A, Henne-Bruns D, Kremer B, Schmiegel W, Neumaier M, Wagener C, Schreiber HW and Kalthoff H (1994) Immunocytological detection of micrometastatic cells: comparative evaluation of findings in the peritoneal cavity and the bone marrow of gastric, colorectal and pancreatic cancer patients. *Int J Cancer* **57**: 330–335

Lindemann F, Schlimock G, Dirschedl P, Witte J and Riethmüller G (1992) Prognostic significance of micrometastatic tumour cells in bone marrow of colorectal cancer patients. *Lancet* **340**: 685–689

Litle VR, Warren RS, Moore II D and Pallavicini MG (1997) Molecular cytogenetic analysis of cytokeratin 20-labeled cells in primary tumours and bone marrow aspirates from colorectal carcinoma patients. *Cancer* **79**: 1664–1670

Long PP, Hruban RH, Lo R, Yeo CJ, Morsberger LA and Griffin CA (1994) Chromosome analysis of nine endocrine neoplasms of the pancreas. *Cancer Genet Cytogenet* **77**: 55–59

Ott G, Kalla J, Ott MM, Schryen B, Katzenberger T, Müller JG and Müller-Hermelink HK (1997) Blastoid variants of mantle cell lymphoma: frequent bcl-1 rearrangements at the major translocation cluster region and tetraploid chromosome clones. *Blood* **89**: 1421–1429

Roka S, Fiegl M, Zojer N, Filipits M, Schuster R, Steiner B, Jakesz R, Huber H and Drach J (1998) Aneuploidy of chromosome 8 as detected by interphase fluorescence in situ hybridization is a recurrent finding in primary and metastatic breast cancer. *Breast Cancer Res Treat* (in press.)

Sakorafas GH, Lazaris A, Tsiotou AG, Koullias G, Glinatsis MT and Golematis BC (1995) Oncogenes in cancer of the pancreas. *Eur J Surg Oncol* **21**: 251–253

Scappaticci S, Brandi ML, Capra E, Cortinovis M, Maraschio P and Fraccaro M (1992) Cytogenetics of multiple endocrine neoplasia syndrome. II. Chromosome abnormalities in an insulinoma and a glucagonoma from two subjects with MEN1. *Cancer Genet Cytogenet* **63**: 17–21

Schenk T, Angerler J, Brunner C, Schenk P, Fiegl M, Zojer N, Huber H and Drach J (1997) Detection of chromosomal aneuploidy by interphase fluorescence in situ hybridization in bronchoscopically gained cells from lung cancer patients. *Chest* **111**: 1691–1696

Soder AI, Hopman AHN, Ramaekers FCS, Conradt C and Bosch FX (1995) Distinct nonrandom patterns of chromosomal aberrations in the progression of squamous cell carcinomas of the head and neck. *Cancer Res* **55**: 5030–5037

Solinas-Toldo S, Wallrapp C, Müller-Pillasch F, Bentz M, Gress T and Lichter P (1996) Mapping of chromosomal imbalances in pancreatic carcinoma by comparative genomic hybridisation. *Cancer Res* **56**: 3803–3807

Teyssier JR (1987) Nonrandom chromosomal changes in human solid tumors: application of an improved culture method. *J Natl Cancer Inst* **79**: 1189–1198

Van der Riet-Fox MF, Retief AE and Van Niekerk WA (1979) Chromosome changes in 17 human neoplasms studied with banding. *Cancer* **44**: 2108–2119

Wiley J, Posekany K, Riley R, Holbrook T, Silverman J, Joshi V and Bowyer S (1995) Cytogenetic and flow cytometric analysis of a pancreatoblastoma. *Cancer Genet Cytogenet* **79**: 115–118

Yamada H, Sakamoto H, Taira M, Nishimura S, Shimosata Y, Terada M and Sugimura T (1986) Amplifications of both c-Ki-ras with a point mutation and c-myc in a primary pancreatic cancer and its metastatic tumors in lymph nodes. *Jpn J Cancer Res* **77**: 370–375

Zojer N, Fiegl M, Angerler J, Müllauer L, Gsur A, Roka S, Pecherstorfer M, Huber H and Drach J (1997) Interphase fluorescence in situ hybridisation improves the detection of malignant cells in effusions from breast cancer patients. *Br J Cancer* **75**: 403–407

© Cancer Research Campaign 1998


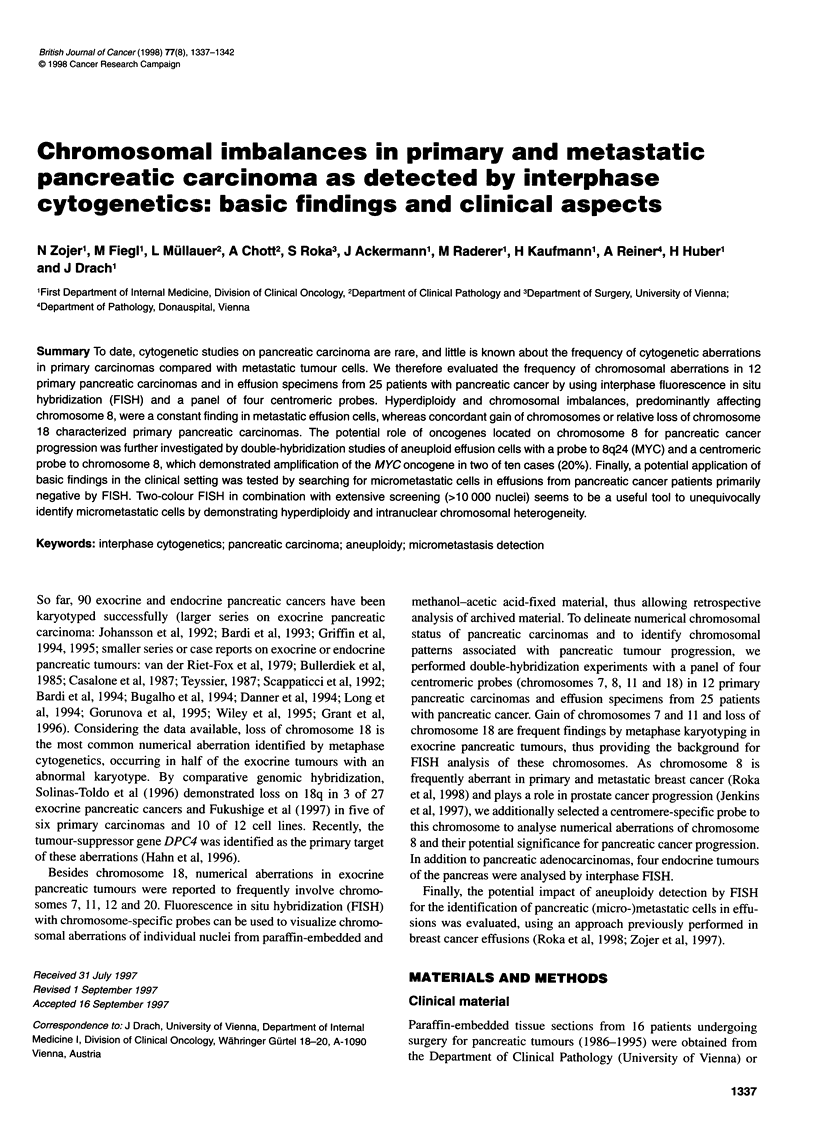

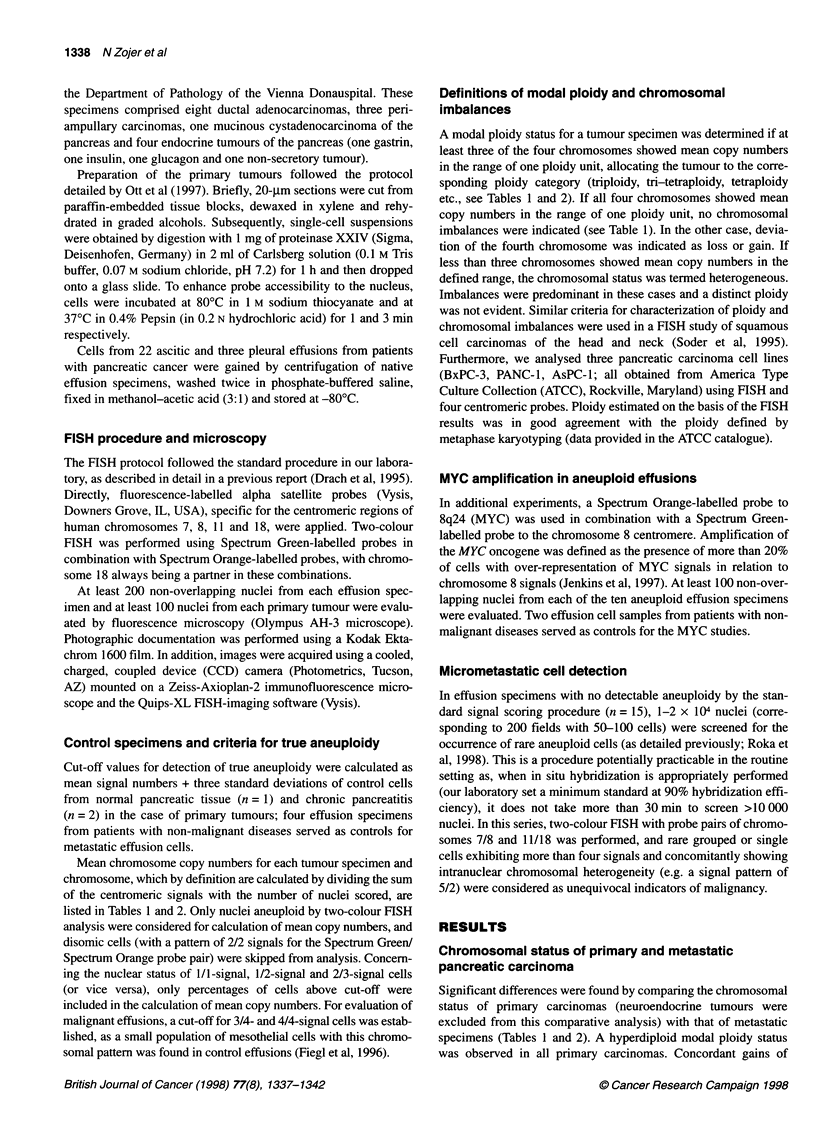

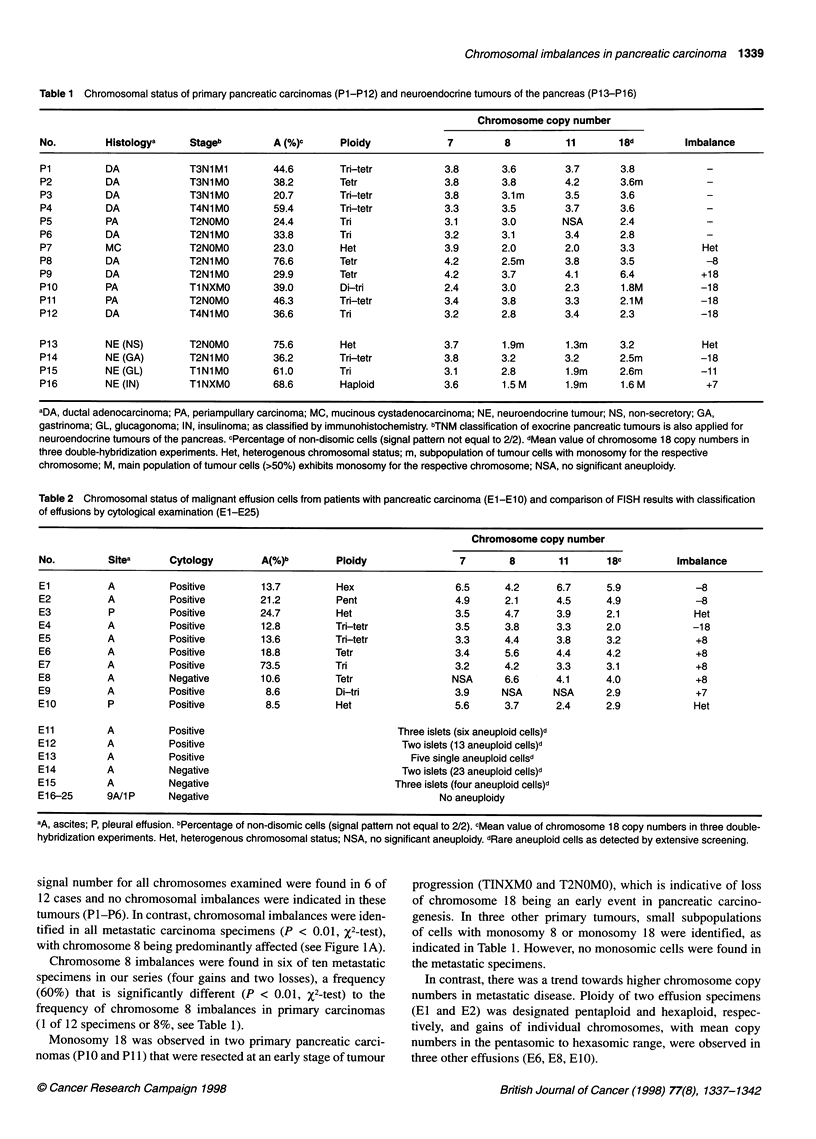

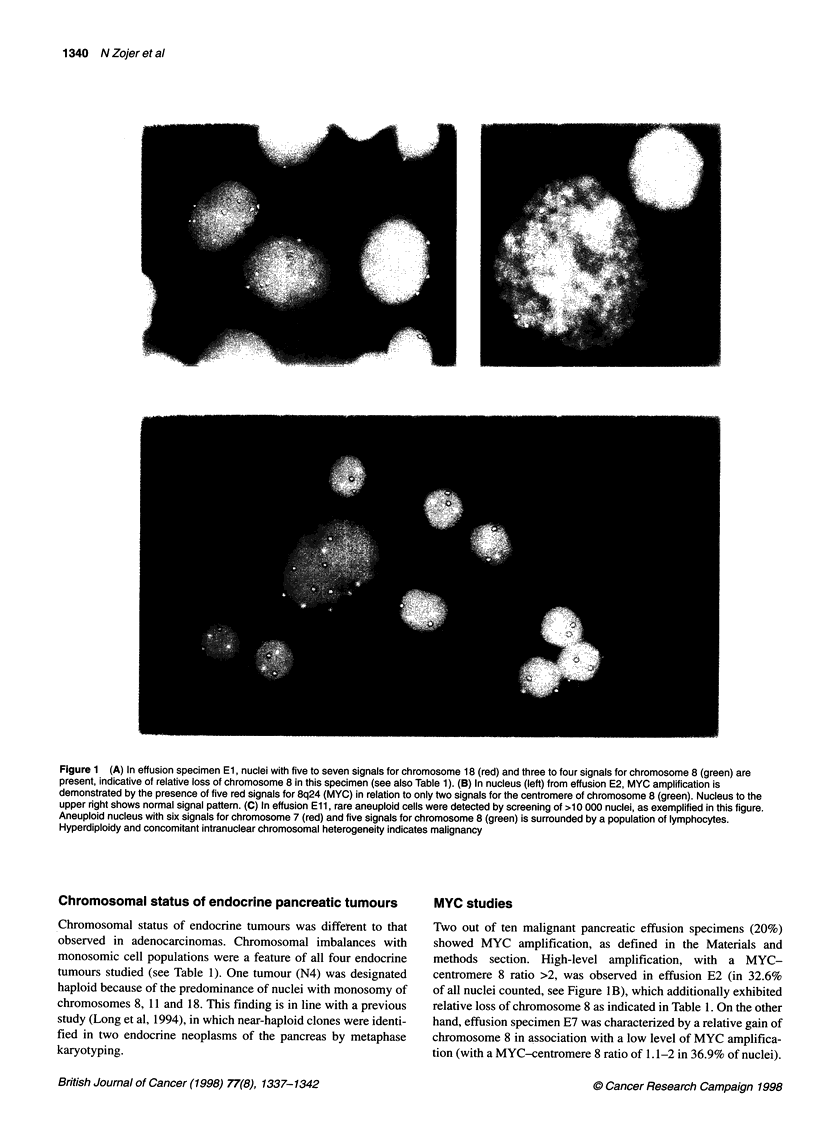

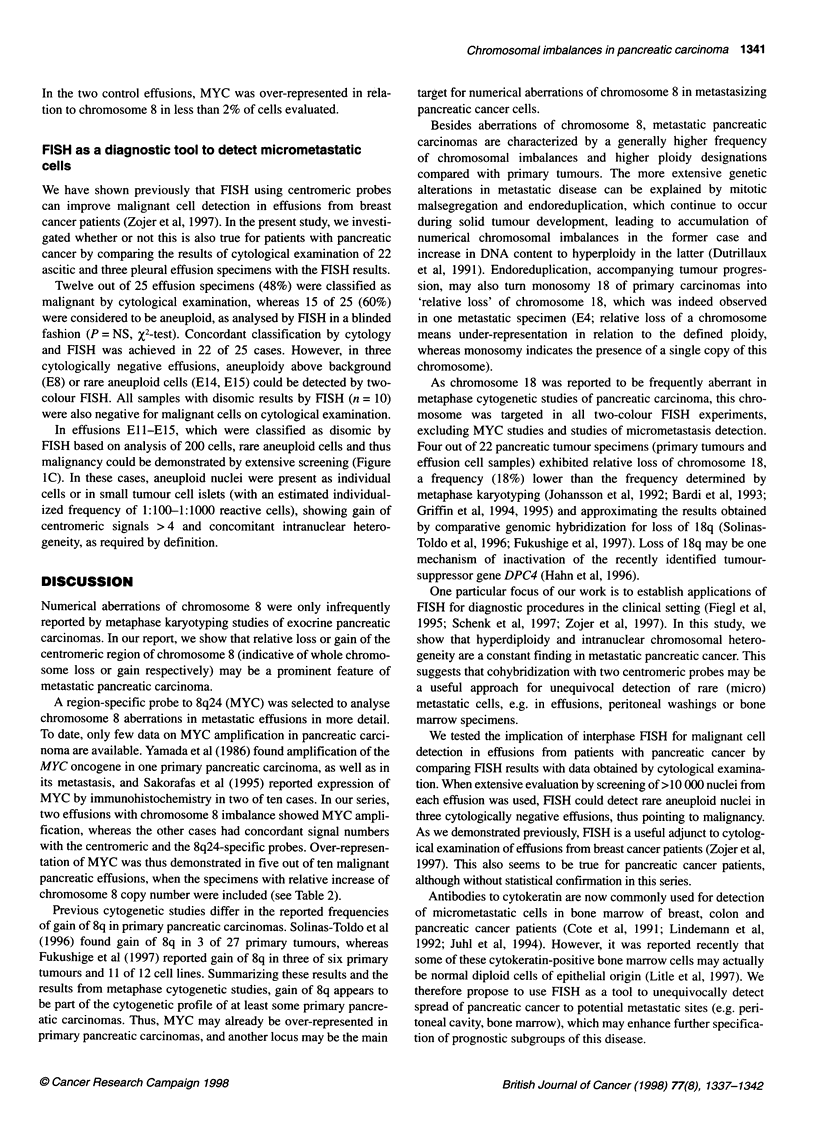

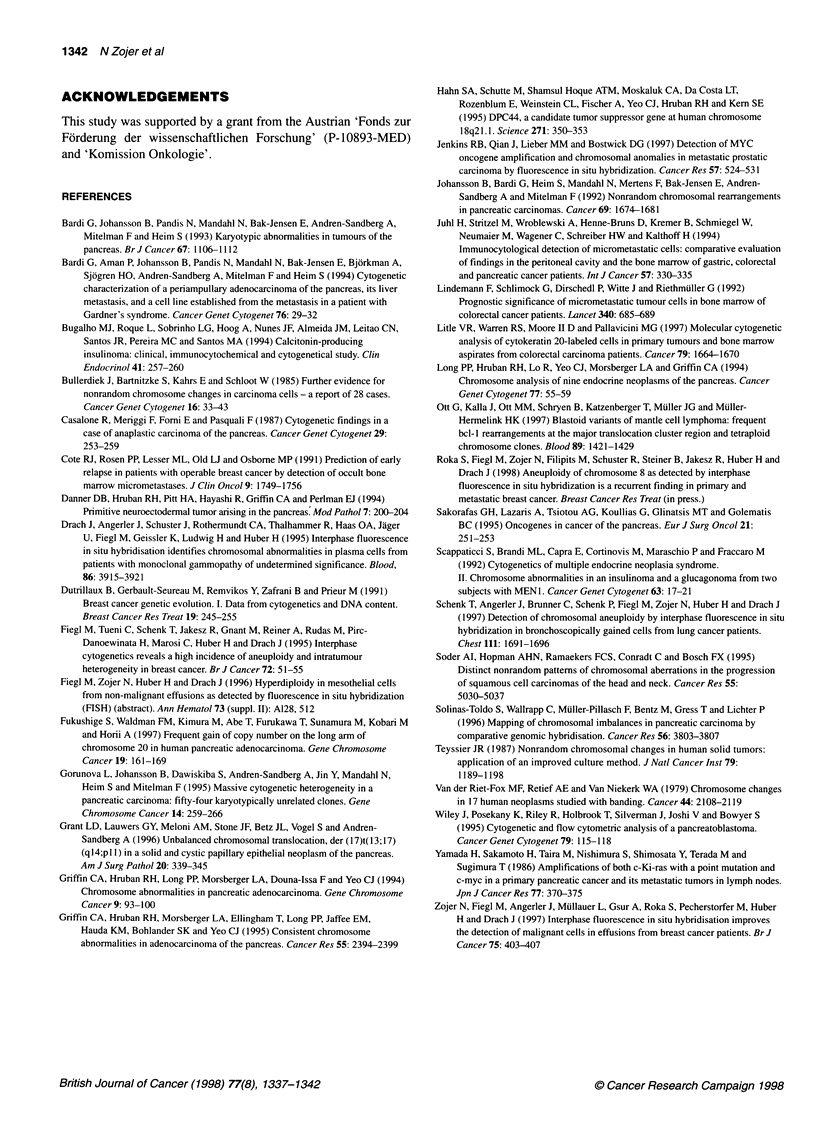


## References

[OCR_00517] Bardi G., Aman P., Johansson B., Pandis N., Mandahl N., Bak-Jensen E., Björkman A., Sjögren H. O., Andrén-Sandberg A., Mitelman F. (1994). Cytogenetic characterization of a periampullary adenocarcinoma of the pancreas, its liver metastasis, and a cell line established from the metastasis and a cell line established from the metastasis in a patient with Gardner's syndrome.. Cancer Genet Cytogenet.

[OCR_00512] Bardi G., Johansson B., Pandis N., Mandahl N., Bak-Jensen E., Andrén-Sandberg A., Mitelman F., Heim S. (1993). Karyotypic abnormalities in tumours of the pancreas.. Br J Cancer.

[OCR_00524] Bugalho M. J., Roque L., Sobrinho L. G., Hoog A., Nunes J. F., Almeida J. M., Leitão C. N., Santos J. R., Pereira M. C., Santos M. A. (1994). Calcitonin-producing insulinoma: clinical, immunocytochemical and cytogenetical study.. Clin Endocrinol (Oxf).

[OCR_00531] Bullerdiek J., Bartnitzke S., Kahrs E., Schloot W. (1985). Further evidence for nonrandom chromosome changes in carcinoma cells--a report of 28 cases.. Cancer Genet Cytogenet.

[OCR_00536] Casalone R., Meriggi F., Forni E., Pasquali F. (1987). Cytogenetic findings in a case of anaplastic carcinoma of the pancreas.. Cancer Genet Cytogenet.

[OCR_00541] Cote R. J., Rosen P. P., Lesser M. L., Old L. J., Osborne M. P. (1991). Prediction of early relapse in patients with operable breast cancer by detection of occult bone marrow micrometastases.. J Clin Oncol.

[OCR_00546] Danner D. B., Hruban R. H., Pitt H. A., Hayashi R., Griffin C. A., Perlman E. J. (1994). Primitive neuroectodermal tumor arising in the pancreas.. Mod Pathol.

[OCR_00549] Drach J., Angerler J., Schuster J., Rothermundt C., Thalhammer R., Haas O. A., Jäger U., Fiegl M., Geissler K., Ludwig H. (1995). Interphase fluorescence in situ hybridization identifies chromosomal abnormalities in plasma cells from patients with monoclonal gammopathy of undetermined significance.. Blood.

[OCR_00556] Dutrillaux B., Gerbault-Seureau M., Remvikos Y., Zafrani B., Prieur M. (1991). Breast cancer genetic evolution: I. Data from cytogenetics and DNA content.. Breast Cancer Res Treat.

[OCR_00563] Fiegl M., Tueni C., Schenk T., Jakesz R., Gnant M., Reiner A., Rudas M., Pirc-Danoewinata H., Marosi C., Huber H. (1995). Interphase cytogenetics reveals a high incidence of aneuploidy and intra-tumour heterogeneity in breast cancer.. Br J Cancer.

[OCR_00573] Fukushige S., Waldman F. M., Kimura M., Abe T., Furukawa T., Sunamura M., Kobari M., Horii A. (1997). Frequent gain of copy number on the long arm of chromosome 20 in human pancreatic adenocarcinoma.. Genes Chromosomes Cancer.

[OCR_00580] Gorunova L., Johansson B., Dawiskiba S., Andrén-Sandberg A., Jin Y., Mandahl N., Heim S., Mitelman F. (1995). Massive cytogenetic heterogeneity in a pancreatic carcinoma: fifty-four karyotypically unrelated clones.. Genes Chromosomes Cancer.

[OCR_00588] Grant L. D., Lauwers G. Y., Meloni A. M., Stone J. F., Betz J. L., Vogel S., Sandberg A. A. (1996). Unbalanced chromosomal translocation, der(17)t(13;17)(q14;p11) in a solid and cystic papillary epithelial neoplasm of the pancreas.. Am J Surg Pathol.

[OCR_00592] Griffin C. A., Hruban R. H., Long P. P., Morsberger L. A., Douna-Issa F., Yeo C. J. (1994). Chromosome abnormalities in pancreatic adenocarcinoma.. Genes Chromosomes Cancer.

[OCR_00597] Griffin C. A., Hruban R. H., Morsberger L. A., Ellingham T., Long P. P., Jaffee E. M., Hauda K. M., Bohlander S. K., Yeo C. J. (1995). Consistent chromosome abnormalities in adenocarcinoma of the pancreas.. Cancer Res.

[OCR_00603] Hahn S. A., Schutte M., Hoque A. T., Moskaluk C. A., da Costa L. T., Rozenblum E., Weinstein C. L., Fischer A., Yeo C. J., Hruban R. H. (1996). DPC4, a candidate tumor suppressor gene at human chromosome 18q21.1.. Science.

[OCR_00609] Jenkins R. B., Qian J., Lieber M. M., Bostwick D. G. (1997). Detection of c-myc oncogene amplification and chromosomal anomalies in metastatic prostatic carcinoma by fluorescence in situ hybridization.. Cancer Res.

[OCR_00615] Johansson B., Bardi G., Heim S., Mandahl N., Mertens F., Bak-Jensen E., Andrén-Sandberg A., Mitelman F. (1992). Nonrandom chromosomal rearrangements in pancreatic carcinomas.. Cancer.

[OCR_00618] Juhl H., Stritzel M., Wroblewski A., Henne-Bruns D., Kremer B., Schmiegel W., Neumaier M., Wagener C., Schreiber H. W., Kalthoff H. (1994). Immunocytological detection of micrometastatic cells: comparative evaluation of findings in the peritoneal cavity and the bone marrow of gastric, colorectal and pancreatic cancer patients.. Int J Cancer.

[OCR_00626] Lindemann F., Schlimok G., Dirschedl P., Witte J., Riethmüller G. (1992). Prognostic significance of micrometastatic tumour cells in bone marrow of colorectal cancer patients.. Lancet.

[OCR_00631] Litle V. R., Warren R. S., Moore D., Pallavicini M. G. (1997). Molecular cytogenetic analysis of cytokeratin 20-labeled cells in primary tumors and bone marrow aspirates from colorectal carcinoma patients.. Cancer.

[OCR_00636] Long P. P., Hruban R. H., Lo R., Yeo C. J., Morsberger L. A., Griffin C. A. (1994). Chromosome analysis of nine endocrine neoplasms of the pancreas.. Cancer Genet Cytogenet.

[OCR_00643] Ott G., Kalla J., Ott M. M., Schryen B., Katzenberger T., Müller J. G., Müller-Hermelink H. K. (1997). Blastoid variants of mantle cell lymphoma: frequent bcl-1 rearrangements at the major translocation cluster region and tetraploid chromosome clones.. Blood.

[OCR_00654] Sakorafas G. H., Lazaris A., Tsiotou A. G., Koullias G., Glinatsis M. T., Golematis B. C. (1995). Oncogenes in cancer of the pancreas.. Eur J Surg Oncol.

[OCR_00659] Scappaticci S., Brandi M. L., Capra E., Cortinovis M., Maraschio P., Fraccaro M. (1992). Cytogenetics of multiple endocrine neoplasia syndrome. II. Chromosome abnormalities in an insulinoma and a glucagonoma from two subjects with MEN1.. Cancer Genet Cytogenet.

[OCR_00666] Schenk T., Ackermann J., Brunner C., Schenk P., Zojer N., Roka S., Drach J. (1997). Detection of chromosomal aneuploidy by interphase fluorescence in situ hybridization in bronchoscopically gained cells from lung cancer patients.. Chest.

[OCR_00672] Soder A. I., Hopman A. H., Ramaekers F. C., Conradt C., Bosch F. X. (1995). Distinct nonrandom patterns of chromosomal aberrations in the progression of squamous cell carcinomas of the head and neck.. Cancer Res.

[OCR_00678] Solinas-Toldo S., Wallrapp C., Müller-Pillasch F., Bentz M., Gress T., Lichter P. (1996). Mapping of chromosomal imbalances in pancreatic carcinoma by comparative genomic hybridization.. Cancer Res.

[OCR_00683] Teyssier J. R. (1987). Nonrandom chromosomal changes in human solid tumors: application of an improved culture method.. J Natl Cancer Inst.

[OCR_00692] Wiley J., Posekany K., Riley R., Holbrook T., Silverman J., Joshi V., Bowyer S. (1995). Cytogenetic and flow cytometric analysis of a pancreatoblastoma.. Cancer Genet Cytogenet.

[OCR_00697] Yamada H., Sakamoto H., Taira M., Nishimura S., Shimosato Y., Terada M., Sugimura T. (1986). Amplifications of both c-Ki-ras with a point mutation and c-myc in a primary pancreatic cancer and its metastatic tumors in lymph nodes.. Jpn J Cancer Res.

[OCR_00704] Zojer N., Fiegl M., Angerler J., Müllauer L., Gsur A., Roka S., Pecherstorfer M., Huber H., Drach J. (1997). Interphase fluorescence in situ hybridization improves the detection of malignant cells in effusions from breast cancer patients.. Br J Cancer.

[OCR_00688] van der Riet-Fox M. F., Retief A. E., van Niekerk W. A. (1979). Chromosome changes in 17 human neoplasms studied with banding.. Cancer.

